# Nurse staffing skill mix and acuity-adjusted provision ratios in Swedish neonatal care: A cohort benchmark study

**DOI:** 10.1016/j.ijnsa.2025.100471

**Published:** 2025-12-16

**Authors:** Pernilla Dillner, Katarina E. Göransson, Mikael Norman, Maria Unbeck, Ulrika Förberg

**Affiliations:** a Department of Neonatology, Karolinska University Hospital, Astrid Lindgren Children’s Hospital, Stockholm, Sweden; b Department of Women’s and Children’s Health, Karolinska Institutet, Stockholm, Sweden; c School of Health and Welfare, Dalarna University, Falun, Sweden; d Department of Clinical Science, Intervention and Technology, Karolinska Institutet, Stockholm, Sweden; e Department of Clinical Sciences, Danderyd Hospital, Karolinska Institutet, Stockholm, Sweden

**Keywords:** Nursing staff, Personnel staffing and scheduling, Intensive care, Neonatal, Patient acuity, Infant

## Abstract

**Background:**

The development of neonatal intensive care has substantially reduced infant mortality, still, infants remain at high risk for adverse outcomes. Safe care relies on adequate nurse staffing and an appropriate skill mix, which is especially important in neonatal intensive care as infants are extremely vulnerable to harm when quality lapses occur. Although international guidelines recommend optimal nurse-to-patient ratios for neonatal care, these standards have not been fully implemented, leaving it unclear whether current staffing levels align with recommendations for safe staffing.

**Objective:**

To benchmark the acuity-adjusted registered nurse staffing provision ratio in neonatal intensive care and determine the skill mix distribution and variation of nursing staff across shifts.

**Method:**

This retrospective cohort study included infant data from a 16-week period in 2022 in three neonatal intensive care units with a common administration at a university hospital. Data were obtained from the hospital’s data repository and the Swedish Neonatal Quality Register, including 609 neonatal admissions and 345 nursing staff members working 1008 shifts. Infants’ daily acuity levels were assessed using an adapted version of the British Association of Perinatal Medicine’s guideline, classifying infants into three levels: intensive care, high dependency care, and special care. Staffing provision was measured as the number of worked hours per shift, staff category, and unit. The registered nurse provision ratio was defined as the number of registered nurse hours provided divided by the recommended hours. A ratio below 1.0 indicates understaffing.

**Results:**

The population’s total in-hospital days were 4674, and the mean birth weight was 2843 g (SD 1029), with 57.0 % being boys. The proportion of registered nurses relative to nursing assistants ranged from 22.2 % to 85.7 %, with a median of 46.5 %. Registered nurses with specialist education accounted for a median of 73.0 % of total registered nurse hours. Within each unit, the mean acuity-adjusted number of registered nurses recommended by the British Association of Perinatal Medicine’s standard was relatively consistent across shift types and between weekdays and weekends. However, the required number of registered nurses between individual shifts showed considerable variation, ranging from 2.5 to 10.3. During the inclusion period, 81.2 % of the shifts had a registered nurse provision ratio below 1.0, suggesting that most shifts did not meet the recommendations for staffing levels.

**Conclusions:**

This study highlights a shortfall in registered nurse staffing relative to recommended levels. Ensuring adequate registered nurse staffing levels is crucial for maintaining high-quality neonatal care and improving infant outcomes.

**What is already known**

• Research findings suggest that hospitals with a higher ratio of registered nurses to less educated nursing staff have lower mortality rates, higher patient satisfaction, and fewer adverse events.
• Neonatal nursing requires registered nurses with specialised competencies to meet the complex medical and emotional needs of neonates and their families.

Alt-text: Unlabelled box

**What this paper adds**

• Staffing levels for registered nurses frequently fell short of recommended standards and a large share of direct patient care was provided by nursing assistants educated at the upper secondary level.
• Requirements for registered nurses were generally consistent across shift types and between weekdays and weekends but varied considerably for individual shifts.
• Specialist-educated registered nurses were statistically significantly more present during night shifts than day shifts.

Alt-text: Unlabelled box

## 1 Background

Effective and high-quality neonatal intensive care has substantially reduced infant mortality rates (World Health Organization, 2024). However, many preterm infants born before 37 weeks of gestation experience short- and long-term adverse outcomes. Infants in neonatal intensive care units (NICUs) are particularly vulnerable to harm from lapses in quality or safety, which optimal care can mitigate (The European Foundation for the Care of Newborn Infants, 2018). Although Sweden is considered to be at the forefront in terms of maternal and neonatal care (Norman, Hallberg, et al., 2019; Zaigham et al., 2024), there is room for improvements, and conditions vary across the country (Swedish National Board of Health and Welfare, 2021).

Nurses play a critical role in patient safety since they provide continuous bedside care and act as the central link in the healthcare team. Nurses monitor for clinical changes, detect errors, and coordinate timely communication across disciplines. Their role encompasses surveillance, early intervention, and ensuring that care processes function effectively (Phillips et al., 2021). Insufficient nurse staffing levels, forces prioritisation of tasks, limiting time for comprehensive care and skilled monitoring. This constraint increases the risk of missed care and, consequently, clinical deterioration (Griffiths et al., 2018).

Neonatal nursing is a specialised field that requires NICU nurses to acquire specific competencies to address the complex needs of neonates and their families (Rogowski et al., 2015; Talus et al., 2023). Consequently, nurse competence is an important factor influencing neonatal outcomes (Hamilton et al., 2007). There is substantial and consistent evidence that insufficient nurse staffing adversely affects patients’ health outcomes (Aiken et al., 2014; Dall'Ora et al., 2022). Research links insufficient nurse staffing levels with higher neonatal mortality (Genna et al., 2023) and increased neonatal morbidities, such as nosocomial infections (Lee et al., 2020; Rogowski et al., 2013).

Optimal nurse staffing levels vary between units and are influenced by multiple interconnected factors. These include patient-related aspects such as patient volume and patient acuity, where acuity refers to the level of nursing care required based on illness severity. In addition, unit-related factors like the availability of support services, the frequency of admissions and discharges, and the unit’s overall structure and resource capacity play a role. Furthermore, nursing-related factors such as staff education, experience, and the skill mix of the nursing team are important in determining appropriate staffing (Feldman and Rohan, 2022).

Various frameworks have been published by professional associations and international organisations to outline optimal and safe registered nurse staffing standards for neonatal care (Association of Women’s Health Obstetric and Neonatal Nurses, 2022; National Association of Neonatal Nurses, 2021; Royal College of Nursing, 2012; Stark et al., 2023; The European Standards of Care for Newborn Health, 2018; World Health Organization, 2017). These frameworks aim to improve patient outcomes and establish safer work environments for registered nurses. However, setting fixed, standard infant-to-registered ratios in NICUs is challenging since infant acuity can vary considerably over time (Gagliardi et al., 2016) and staffing levels need to align with the specific nursing care needs of infants (Kane et al., 2007; Rogowski et al., 2015).

Acuity classification can assist in evaluating whether registered nurse staffing is sufficient by identifying the registered nurse effort necessary to provide safe and effective care according to the infant’s care needs (Association of Women’s Health Obstetric and Neonatal Nurses, 2022). The British Association of Perinatal Medicine (The British Association of Perinatal Medicine, 2011) has established a patient classification system categorising infants into three acuity categories: intensive care, high-dependency care and specialist care. A similar system by the American Academy of Pediatrics and the American College of Obstetricians and Gynecologists (Rogowski et al., 2015) defines five levels of infant acuity, with the key difference being further subdivisions of intensive care and special care infants.

The British Association of Perinatal Medicines staffing guidelines (The British Association of Perinatal Medicine, 2022) specify neonatal infant-to-registered nurse ratio based on infant acuity level. According to these guidelines, the minimum staffing requirements are one registered nurse per intensive care infant (1:1), one registered nurse for every two high dependency care infants (1:2), and one registered nurse for every four specialist care infants (1:4). Each shift should also include a coordinator, typically a registered nurse with extensive neonatal care experience. Similar staffing recommendations are outlined by the Association of Women's Health, Obstetric and Neonatal Nurses, ranging from one registered nurse per critically ill infant (1:1) to one registered nurse for every five newborns needing routine care (1:5) (Association of Women’s Health Obstetric and Neonatal Nurses, 2022).

The Swedish National Board of Health and Welfare emphasises the importance of adequate staffing levels to ensure the delivery of high-quality care. However, the absence of specific recommendations regarding patient-to-registered nurse ratios means that staffing levels are typically determined by local healthcare providers (Swedish National Board of Health and Welfare, 2018). This variability can lead to inconsistencies in care quality across different units and individual shifts. Given the critical role of registered nurses in NICUs and the potential for adverse outcomes due to inadequate staffing, this study aims to benchmark the acuity-adjusted registered nurse staffing provision ratio in neonatal intensive care and determine the skill mix distribution and variation of nursing staff across shifts.

## 2 Methods

### 2.1 Study design and setting

This retrospective cohort study included infant data collected over a 16-week period, from 7 March 2022 to 26 June 2022. It involved three NICUs administered by Karolinska University Hospital, each located at a different site. The three units were selected because they provided complete and accessible data relevant to the research question, making them suitable for inclusion in the study.

In Sweden, neonatal care is delivered at 38 hospitals. Eight hospitals provide full neonatal intensive care (levels III– IV), while 27 hospitals offer partial neonatal intensive care (level II). In addition, three hospitals provide care only for near-term and full-term newborns (level I). Approximately one in ten births results in a newborn requiring NICU care, totalling 10,875 admissions in 2022, with about one-third due to preterm birth (Swedish Neonatal Quality Register, 2022).

This study included one Level 4 NICU, which provided care for the most medically complex and critically ill infants from 22 weeks of gestation, including those requiring neonatal surgery; one Level 3 NICU, which provided care from 26 weeks of gestation and specialised in neonatal dialysis; and one Level 2 NICU, which managed stable or moderately ill infants from 32 weeks of gestation. At the time of the study, the NICUs had a combined capacity of approximately 40 inpatient beds, 40 beds for hospital-assisted home care and a workforce of around 450 healthcare professionals, supporting approximately 26,000 annual deliveries.

NICU nurses in Sweden have varying levels of education and expertise. While there are local and regional educational programmes in neonatal nursing, there is no formal national speciality. The NICU nursing team consists of registered nurses, with a proportion holding specialist education and nursing assistants. Registered nurses obtain a Bachelor of Science in Nursing after completing a three-year programme. Registered nurses with specialist education hold a speciality certification in fields such as paediatrics, midwifery or intensive care, following a one- or one-and-a-half-year Master of Science in Nursing programme. Nursing assistants require education at the upper secondary level or equivalent.

### 2.2 Participants

All neonatal infants admitted to and present at the three NICUs during the 16-week inclusion period were included. Similarly, all nursing staff members (registered nurses, registered nurses with specialist education and nursing assistants) providing clinical care during this period were part of the study sample. Nonclinical staff hours, including time spent on education, administrative duties, staff under introduction and students, were excluded. Shift coordinators, typically registered nurses with specialist education, were included separately, as they primarily managed infant transfers and served as communication links between staff, managers and physicians within the hospital and at transfer hospitals.

### 2.3 Data sources and data collection

Patient data were obtained from the hospital’s data repository and the Swedish Neonatal Quality Register (Norman, Källén, et al., 2019). The hospital data encompassed details on the unit, patient ID, admission and discharge dates and times, transfers, home leaves, main and secondary diagnoses and procedure codes. The infant’s length of stay was summarised in hours present per shift. Daily reports for each infant were extracted from the Swedish Neonatal Quality Register, covering 26 different interventions, including mechanical respiratory support, umbilical lines, chest drains, invasive respiratory support and parenteral nutrition, among others.

The infants’ acuity category per day was assessed using an adapted version of the British Association of Perinatal Medicines classification (The British Association of Perinatal Medicine, 2011), derived from data provided by the Swedish Neonatal Quality Register (Norman, Källén, et al., 2019). In our study, infants receiving parenteral nutrition but not requiring respiratory support were classified as high-dependency care infants instead of intensive care infants, as defined by the original British Association of Perinatal Medicines classification (supplementary material Table S1). We determined that some of these patients might belong to the intensive care category, although most would not require intensive care. This adjustment was made to avoid categorising infants at a higher acuity level than necessary, thereby reducing the number of falsely low registered nurse provision ratios. The assessed acuity category, according to the adapted British Association of Perinatal Medicines classification, was recorded in a Microsoft Excel spreadsheet, with the acuity category noted for each day for every admitted infant.

Staffing data were obtained from the hospital’s Human Resource Management System (Heroma™), while agency staffing shifts were sourced separately from Pibook™ (the staffing agency statistics system). Daily written staffing reports were also collected to allow for manual verification of any potential discrepancies. Staffing data validity was ensured through repeated meetings with staffing assistants during the data collection period. The staffing data included individual shift start and end times, work activities (e.g., clinical, administrative and educational), and levels of education for all staff categories employed by the hospital during the 16-week inclusion period at the three NICUs.

The included NICUs operated on a three-shift staffing structure: day (07:00–15:30), evening (13:30–21:30) and night (21:00–07:15). During shift overlaps, staff from the upcoming shift were not included in the staffing numbers. Thus, day shift staff members were included between 07:00 and 14:00 (7 h), evening shifts between 14:00 and 21:00 (7 h) and night shifts between 21:00 and 07:00 (10 h). Due to substantial variations in shift patterns, with individual staffing shift durations ranging from 4 to 15 h, staffing provision per shift was summarised as hours present for each staff category, shift and NICU. The 24-hour cutoff was set at 7:00 for both staff and infants to align with the night shift end time. Night shift staffing was attributed to the date the shift started, and infants’ admission times between 0:00–06:59 were counted as part of the night shift from the previous date.

### 2.4 Statistical analysis

Frequencies, percentages, means (standard deviation, 95 % CI) and medians (interquartile range) were used for descriptive statistics. Staffing provision was measured by the number of worked hours per shift, NICU and staff category (registered nurses, registered nurses with specialist education and nursing assistants). The recommended number of registered nurse hours per shift and NICU was calculated by multiplying the number of infant hours per acuity category by 1.0 for intensive care, by 0.5 for high-dependency care and by 0.25 for special care, according to the recommendations set forth by the British Association of Perinatal Medicine

(The British Association of Perinatal Medicine, 2022) (supplementary material Table S2). The registered nurse provision ratio was defined as the provided number of registered nurse hours divided by the required number of registered nurse hours per shift and NICU. A registered nurse provision ratio of less than 1.0 indicates understaffing. The registered nurse and registered nurse with specialist education staff hours were combined when calculating the registered nurse provision ratio, while nursing assistants were excluded from the calculations. In this study, weekends were defined as Saturdays and Sundays. The Kruskal–Wallis test was used to compare levels of registered nurses with specialist education between shift types within NICUs. A Mann–Whitney U test was performed to compare the registered nurse provision ratio across shift types between weekdays and weekends. The p-value was set to 0.05. Statistical analyses were conducted using SPSS Statistics, version 29.0 (IBM Corp.). Data visualisations were created using the ggplot2 package (version 3.5.1) in R Software (version 4.4.1) (R Foundation for Statistical Computing, Vienna, Austria).

### 2.5 Ethical considerations

The research has followed national and international ethical principles. This study was approved by the Swedish Ethical Review Authority (Dnr. 2021–04,962 and 2024–02,426–02). Consent has not been obtained from each participant. The head of each department and the Swedish Neonatal Quality Register granted permission for data access in accordance with the ethical approval.

## 3 Results

### 3.1 Study population and staffing characteristics

During the 16-week inclusion period, 609 neonatal admissions (535 unique infants) were cared for across three NICUs by a team of 345 nursing personnel, including registered nurses, registered nurses with specialist education and nursing assistants. The included infants had a mean gestational age of 36.6 weeks (SD 4.3) and a mean birth weight of 2843 g (SD 1029). The infants’ lengths of stay averaged *10.4 days (SD 16.9), with a median of 4.0* days (IQR 8) (Table 1). In total, nursing staff worked 79,799.9 h across 1008 shifts, with an equal distribution across day, evening and night shifts. On average, the hours worked per shift for each staff category were 11.6 (SD 7.6) for registered nurses, 25.1 (SD 12.6) for registered nurses with specialist education, and 43.4 (SD 12.5) for nursing assistants (Table 2). A designated coordinator was present during 85.4 % of day shifts but only 1.2 % of evening shifts and was absent during night shifts. An overview of the worked hours related to all nursing staff per unit, shift and staff category can be seen in the supplementary material Table S3.

**Table 1:** Infant demographics.

Infants	Unique infants (*n* = 535)	Admissions (*n* = 609)
Gender, male, n (%)	305 (57.0)	348 (57.1)
GA (weeks), mean (SD)	36.6 (4.3)	36.1 (4.5)
GA (weeks), n (%)		
23−28	34 (6.4)	48 (7.9)
28−32	44 (8.2)	61 (10.0)
32–37	153 (28.6)	177 (29.1)
>37	304 (56.8)	323 (53.0)
Birth weight (g), mean (SD)	2843 (1029)	2747 (1061)
Birth weight (g), n (%)		
≤1000	32 (6.0)	45 (7.4)
1001–2500	155 (29.0)	193 (31.7)
> 2500	348 (65.0)	371 (60.9)
In-hospital days, n	4674	4674
LOS^a,^^b^ days, median (IQR)	4.0 (8)	4.0 (8)
LOS^a,^^b^ days/GA (weeks), mean (SD)	10.4 (16.9)	9.8 (14.2)
23−28	52.6 (33.6)	37.3 (29.5)
28−32	27.2 (18.6)	19.6 (15.3)
32–37	9.5 (7.9)	8.2 (6.7)
>37	3.8 (3.9)	3.6 (3.5)

Note: ^a^ Full admission of included infant, not limited to the inclusion period.

b Length of stay was calculated as (date of discharge – date of admission +1). Abbreviations: LOS, length of stay; GA, gestational age.

**Table 2:** Staffing characteristics.

Variable	Value
Registered nurses, n (%)	54 (15.7)
Specialist nurses, n (%)	103 (29.5)
Nursing assistants, n (%)	188 (54.5)
**Total number of staff members (%)**	**345 (100.0)**
Registered nurses, mean (SD) worked hours per shift	11.6 (7.6)
Specialist nurses, mean (SD) worked hours per shift	25.1 (12.6)
Nursing assistants, mean (SD) worked hours per shift	42.4 (12.5)
**Total number of worked hours**	**79,799.9**

Note: Total number of shifts = 1008. Specialist nurses refers to registered nurses with specialist education.

### 3.2 Nursing skill mix

**Fig. 1:**
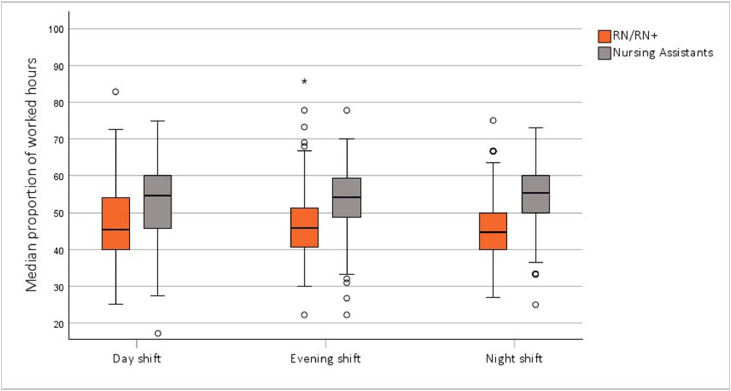
Distribution of staff hours by staff category and shift type.

Note: Circles (o) denote mild outliers, and stars (*) represent extreme outliers. Abbreviations: RN, registered nurse; RN+, registered nurse with specialist education.

The median proportions of registered nurses (including registered nurses with specialist education) and nursing assistants remained relatively consistent across day, evening and night shifts, with registered nurses accounting for 46.5 % of total staffing hours and nursing assistants for 53.5 %. However, there was notable variation within shifts. During day shifts, the proportion of registered nurse hours ranged from 25.0 % to 82.8 %, during evening shifts from 22.2 % to 85.7 %, and during night shifts from 27.0 % to 75.0 % (Fig. 1). The median proportion of registered nurse hours was similar on weekdays and weekends (supplementary material Fig. S1).

When focusing on registered nurses with specialist education, the median proportion of hours worked by these nurses relative to total registered nurse hours, reflecting the skill mix within the workforce, was 73.0 % (IQR 30), with a mean of 66.3 % (SD 23). The highest skill mix was observed during night shifts at 79.8 % (IQR 17), while the lowest occurred during day shifts (median 60.9 %, IQR 27). This proportion varied between NICUs and shift types: the Level 4 NICU had the highest median skill mix (83.2 %, IQR 20) during night shifts, whereas the Level 2 NICU had the lowest (40.0 %, IQR 31) during day shifts (see Supplementary Material Fig. S2). Differences in skill mix were statistically significant between day and night shifts across all NICUs (*p* < 0.001 for Level 2 and Level 4, *p* = 0.009 for Level 3), as well as between evening and night shifts at Level 2 and Level 4 NICUs (*p* < 0.001). Additionally, the Level 2 NICU showed a statistically significant difference between day and evening shifts (*p* = 0.013).

### 3.3 Infant acuity category

**Fig. 2:**
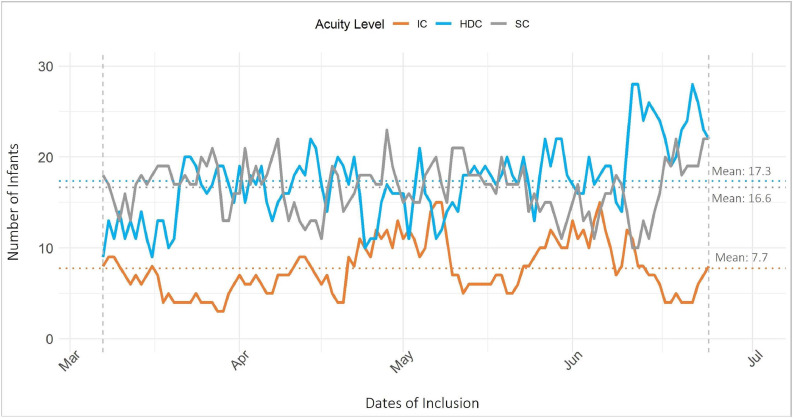
Number of admitted infants per acuity category during the 16-week inclusion period.

Abbreviations: IC, intensive care; HDC, high-dependency care; SC, special care.

Out of the total 4674 in-hospital days, the distribution of acuity was 18.6 % for intensive care infants, 41.6 % for high-dependency care infants and 39.9 % for special care infants. The number of admitted infants per acuity category fluctuated throughout the inclusion period. The number of intensive care infants had daily variations ranging from 3 to 15 (mean 7.7); the number of high-dependency care infants ranged from 9 to 28 (mean 17.3); and the number of special care infants ranged from 10 to 23 (mean 16.6) (Fig. 2).

Insert Fig. 2 (Number of admitted infants per acuity category during the 16-week inclusion period.) here.

In the Level 4 NICU, which accounted for 1238 (26.5 %) in-hospital days, the acuity distribution revealed a high prevalence of intensive care (42.0 %), 47.7 % classified as high-dependency care and 10.3 % classified as special care. The Level 3 NICU, which accounted for 1687 (36.1 %) in-hospital days, had an acuity distribution of 17.1 % intensive care, 41.1 % high-dependency care and 41.8 % special care. The Level 2 NICU, accounting for 1749 (37.4 %) in-hospital days, exhibited a lower acuity level, with 3.3 % classified as intensive care, 37.7 % as high-dependency care and 58.9 % as special care (supplementary material Table S4).

### 3.4 Guideline-based registered nurse staffing recommendations

The results highlight the varying registered nurse requirements across different levels of NICUs, influenced by both the number of infants and their acuity levels. Within each NICU level, the mean registered nurse requirement remained relatively consistent between shift types and between weekdays and weekends (Table 3). However, the required number of registered nurses across individual shifts showed considerable variation. In the Level 4 NICU, registered nurse staffing requirements ranged from 4.5 to 10.1 nurses per shift. Similarly, in the Level 3 NICU, the range was between 3.1 and 10.3 registered nurses, while in the Level 2 NICU, registered nurse requirements varied from 2.5 to 7.0 per shift.

**Table 3:** Acuity-adjusted mean number of registered nurses recommended a per shift and unit.

Shift type	Level 4 NICU mean (95 % CI)	Level 3 NICU mean (95 % CI)	Level 2 NICU mean (95 % CI)
**Mon−Fri** (240 shifts per shift type)			
Day	6.8 (6.5 − 7.1)	6.3 (5.9 − 6.6)	4.7 (4.5 − 4.9)
Evening	6.7 (6.4 − 7.0)	6.3 (6.0 − 6.6)	4.7 (4.5 − 4.9)
Night	6.9 (6.6 − 7.2)	6.4 (6.1 − 6.8)	4.8 (4.6 − 5.0)
**Sat−Sun** (92 shifts per shift−type)			
Day	6.9 (6.4 − 6.9)	6.6 (6.1 − 7.2)	4.7 (4.3 − 5.0)
Evening	6.9 (6.4 − 7.5)	6.6 (6.1 − 7.1)	4.7 (4.2 − 4.9)
Night	7.1 (6.6 − 7.7)	6.8 (6.3 − 7.3)	4.7 (4.4 − 5.0)

Note: Level 4 units provided full intensive care (including neonatal surgery) for the most medically complex and critically ill infants. Level 3 units provided care for medically complex and critically ill infants from gestational week 26. Level 2 units cared for stable or moderately ill infants at ≥32 weeks of gestation.

a RN staffing recommendation from the British Association of Perinatal Medicine (The British Association of Perinatal Medicine, 2022). Abbreviations: NICU, neonatal intensive care unit; RN, registered nurse.

### 3.5 Registered nurse provision ratio

**Fig. 3:**
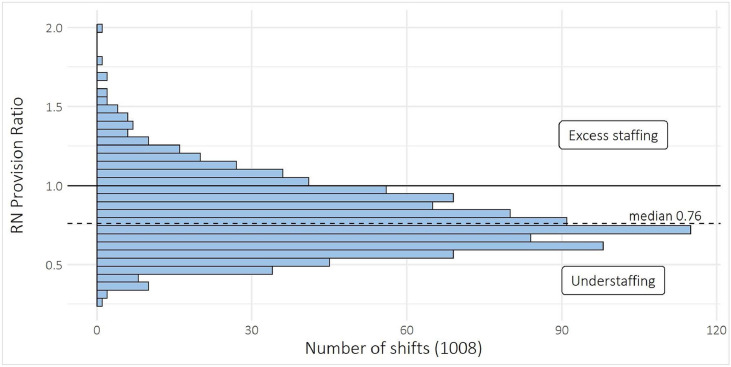
Registered nurse provision ratio.

Note: In total, 1008 shifts. Values below 1.0 indicate a lower-than-recommended presence of registered nurses, as per the British association of perinatal medicines guidelines, relative to infants’ staffing needs. Registered nurse and registered nurse with specialist education staff hours were combined when calculating the registered nurse provision ratio. Abbreviations: RN, registered nurse.

Out of a total of 1008 shifts, 81.2 % had a registered nurse provision ratio below 1.0, suggesting that the majority of shifts did not meet the recommended staffing levels set forth by the British Association of Perinatal Medicine (The British Association of Perinatal Medicine, 2022). The median registered nurse provision ratio was 0.76, with an interquartile range of 0.30, meaning that for 50 % of the shifts, registered nurse provision was 24 % lower than the recommended staffing levels. The ratio ranged from 0.25 to 2.0 across all shifts (Fig. 3).

The data also revealed a statistically significant (*p* = 0.015) reduction in registered nurse provision during day shifts on weekends compared to weekdays. However, registered nurse provision during evening and night shifts remained consistent across weekdays and weekends (supplementary material Table S5).

## 4 Discussion

Scientific evaluation of nurse staffing in NICUs in relation to infant acuity is limited. This study is the first to examine registered nurse provision ratio in relation to infant acuity within Swedish NICUs. The findings raise concerns regarding registered nurse staffing levels across shifts when compared to the staffing recommendations set forth by the British Association of Perinatal Medicines (The British Association of Perinatal Medicine, 2022). A substantial proportion of shifts (81.2 %) had a registered nurse provision ratio below the recommended standard of 1.0, with a median registered nurse provision ratio of 0.76. This suggests that for half of the shifts, there were 24 % fewer registered nurse hours than recommended, pointing to a consistent staffing shortfall that could potentially affect the quality of care provided to infants (Genna et al., 2023). Insufficient staffing of registered nurses is a clear risk factor, as shifts with lower registered nurse-to-patient ratios have been associated with an increased risk of patient mortality (Needleman et al., 2020).

While the unpredictable nature of neonatal care means that meeting recommended staffing levels may not always be feasible, it remains essential that the average registered nurse provision ratio closely aligns with these standards to ensure adequate care. Maintaining a sufficiently high baseline roster is particularly important, as scheduling too few nurses in advance limits the ability to respond to demand fluctuations and may compromise patient safety (Griffiths et al., 2021)

A British study conducted in 2009 found that 54 % of nursing shifts were understaffed according to the British Association of Perinatal Medicines standards. This understaffing led to a 28 % reduction in the time spent on clinical care per infant, and essential tasks were delayed or omitted. Delays or omissions were more likely when staffing levels did not meet the standards (Pillay et al., 2012).

The In this study, a median of 73.0 % (mean 66.3 %) of registered nurse provision hours came from registered nurses with specialist education, in line with the recommended minimum of 70 % set by the British National Health Service for post-registration qualifications (National Health Service and Department of Health, 2009). A recent review on nurse staffing in NICUs underlines that a higher proportion of registered nurses with specialist neonatal qualifications is linked to reduced mortality and morbidity (Genna et al., 2023). However, the skill mix distribution between registered nurses and nursing assistants in this study was 45 % to 55 %. This proportion is lower than the Swedish average of 58 % registered nurses, reported in adult care in 2013, and among the lowest in Europe (Aiken et al., 2013). This suggests that the primary challenge for the NICUs in this study lies not in the total number of nursing staff hours provided, but in the level of education among staff, as a substantial proportion of direct patient care was delivered by nursing assistants with upper secondary-level education.

While nursing assistants are vital in providing essential care, they cannot replace registered nurses (Griffiths et al., 2019). Swedish hospitals, like healthcare systems in many countries, face challenges in recruiting and retaining registered nurses (World Health Organization, 2020). Research has shown that hospitals with a higher proportion of registered nurses, compared to less educated nursing staff, experience lower mortality, higher patient satisfaction and fewer adverse events (Aiken et al., 2017). The contribution of support staff to direct patient care varies globally and is often overlooked in staffing models, particularly those from the United States (Griffiths et al., 2020). In Europe, adjusting the skill mix is a common strategy to address workforce shortages (Aiken et al., 2013). However, relying on a less qualified skill mix not only compromises patient outcomes (Genna et al., 2023) but can also exacerbate registered nurse shortages by limiting training opportunities for registered nurse students and decreasing job satisfaction and retention (Aiken et al., 2002; Jacob et al., 2015).

While there was no notable difference in the average number of registered nurse hours required across shift types or between weekdays and weekends, the demand for registered nurses varied substantially across individual shifts (ranging from 4.5 to 10.3 registered nurses in the Level 4 NICU). This variation was driven by the dynamic care needs of infants, as well as fluctuations in the number of admitted patients. These variations underscore the challenges in ensuring adequate staffing in neonatal care settings, where staffing levels must continuously adapt to changing and often unplanned patient requirements.

Staffing in neonatal care will always be complex. To better understand and manage staffing challenges, patient classification systems have been developed to estimate nursing resource needs by identifying individual patients’ care requirements (Junttila et al., 2016; Roth et al., 2023). In this study, we used an adapted version of a classification system developed by the British Association of Perinatal Medicines (The British Association of Perinatal Medicine, 2011) to assess infants’ acuity. This system has been shown to align well with other similar systems (Gagliardi et al., 2016), such as the one described by the American College of Obstetricians and Gynecologists (Rogowski et al., 2015). We chose to apply the British Association of Perinatal Medicine categorization (The British Association of Perinatal Medicine, 2011), because the available data provided sufficient detail to support classification into three categories. but not into five, as proposed by the American Academy of Pediatrics and the American College of Obstetricians and Gynecologists (Association of Women’s Health Obstetric and Neonatal Nurses, 2022). Although a five-level categorization might have allowed for a more precise estimation of overall care needs per shift, the three-category system was best suited to the level of detail in our dataset.

The proportion of intensive care days varies depending on the type of NICU and the classification system used (Ohnstad and Solberg, 2017). In this study, the distribution of acuity per patient day was 19 % for intensive care infants and 42 % for high-dependency care infants. Without the adaptation of the British Association of Perinatal Medicine´s system, infants who received parenteral nutrition but did not require respiratory support would have been classified as intensive care infants, resulting in a higher proportion (close to 26 %) of intensive care patients in this sample. The relatively low proportion of special care infants compared to other studies (Gagliardi et al., 2016; Ohnstad and Solberg, 2017; Patry et al., 2014), is likely due to the well-established hospital-assisted home care programme, which admits stable infants at 34 weeks of gestation to neonatal home care.

### 4.1 Strengths and limitations

Many studies analysing staffing do not account for day-to-day variations in staffing levels (Genna et al., 2023). In contrast, this study provides detailed data on every infant's admission hour, alongside staffing levels at the same times over the 16 weeks of inclusion. This approach allows for thorough analyses that consider fluctuations in both the infant's condition and the individual presence of staff members, providing a more comprehensive understanding of the relationship between staffing and patient care needs. A key strength of the study is the use of digital personnel data, which have been validated against staffing assistants and manual scheduling records, ensuring high accuracy in staffing records. Additionally, the inclusion of agency staff ensures 100 % coverage of staffing data, offering a complete view. Furthermore, infant acuity was extracted from the well-established Swedish Neonatal Quality Register, which involves daily manual entry of a wide range of parameters from patient records. As a key resource for neonatal care research, Swedish Neonatal Quality Register is known for its comprehensive data and reliable tracking of neonatal care, contributing to the robustness of the acuity information (Norman, Källén, et al., 2019).

The British Association of Perinatal Medicine’s patient classification system categorises infants based on the level of medical treatment required. Defining optimal staffing levels is challenging, as infant acuity is one key factor. Other considerations, such as time spent supporting parents in crisis, as well as factors like infant admissions, discharges, and transfers, all contribute to nursing workload (Simpson, 2021). Additionally, staffing needs can be influenced by staff productivity, team experience, individual nurse performance, and unit-specific elements like team dynamics and the physical layout of the unit (Griffiths et al., 2020; Rogowski et al., 2015).

It is possible that a system which bases nurse workload solely on the infants’ medical needs may undervalue the staffing requirements for example of a NICU with a lower proportion of critically ill infants, but which may have other needs that require resources, such as parental support and high level of admissions and discharges. Relying solely on patient acuity or severity of illness may not provide a complete picture (Feldman and Rohan, 2022; Griffiths et al., 2020; Rogowski et al., 2015).

Different approaches to staffing tools in healthcare are designed to estimate the number of staff needed to provide safe and effective patient care. These approaches vary in their methodologies and the factors they consider. A challenge with many patient classification systems is the lack of a strong scientific foundation, including consistent testing for validity and reliability, as well as limited evidence linking them to nurse-sensitive outcomes (Griffiths et al., 2020). The care provided and staffing levels can vary between hospitals and countries, which may affect the generalisability of the findings in this study.

### 4.2 Clinical implications

Measuring patient needs by shift is useful for guiding flexible staff deployment. Despite the ongoing need for accurate information, structural barriers often hinder data collection in healthcare settings (Feldman and Rohan, 2022). However, simply collecting data is insufficient to improve nurse staffing; professional judgment, which draws on healthcare professionals’ expertise and experience, is crucial in developing and evaluating staffing plans (Griffiths et al., 2021). While professional judgment provides valuable contextual insights—such as team dynamics or unforeseen events—it is essential that it is supported by data-generated information. The data serve as a foundation, ensuring that decisions are grounded in measurable factors, while professional judgment adds the nuance and flexibility needed to adapt to changing circumstances. Future studies should focus on overcoming structural barriers to collecting and operationalising data in healthcare settings, particularly in NICUs. Establishing clear staffing guidelines can help create a safer and more supportive work environment for registered nurses, reducing burnout and improving job satisfaction. The findings of this study highlight the need for national and local policies on registered nurse staffing in NICUs, leading to more consistent and high-quality care.

## 5 Conclusions

This study provides the first report on nurse staffing in Swedish NICUs in relation to infant acuity. The findings underscore substantial deficiencies in registered nurse staffing levels, as a sizable proportion of shifts does not meet the recommended staffing ratios. Furthermore, the skill-mix within the workforce, with a notable share of nursing assistants, presents a key challenge in ensuring optimal care delivery. By aligning staffing levels with the specific needs of infants, healthcare providers can deliver more effective and individualised care, leading to better short- and long-term outcomes.

## Funding statement

This work was supported by grants from H.K.H. Crown Princess Lovisa Association (award number 2024–026), Löf (the Swedish National Patient Insurance Company), the Samariten Foundation for Paediatric Research and Sällskapet Barnavård Foundation (The Society for Child Welfare). The funders of the study had no role in the study design, data collection, data analysis, data interpretation or manuscript writing.

## Data availability

The data used to support the findings of this study are available from the corresponding author upon reasonable request.

## CRediT authorship contribution statement

**Pernilla Dillner:** Writing – review & editing, Writing – original draft, Visualization, Project administration, Methodology, Investigation, Formal analysis, Data curation, Conceptualization. **Katarina E. Göransson:** Writing – review & editing, Supervision, Methodology, Investigation, Conceptualization. **Mikael Norman:** Writing – review & editing, Conceptualization. **Maria Unbeck:** Writing – review & editing, Conceptualization. **Ulrika Förberg:** Writing – review & editing, Supervision, Methodology, Investigation, Conceptualization.

## Declaration of competing interest

The authors declare no conflicts of interest.

## Appendix Supplementary materials

Image, application 1
