# Supplementary material for: Nurse staffing skill mix and acuity-adjusted provision ratios in Swedish neonatal care: A cohort benchmark study
Source: Int J Nurs Stud Adv. 2025 Dec 16;10:100471. doi: 10.1016/j.ijnsa.2025.100471 (PMC12808838; doi:10.1016/j.ijnsa.2025.100471)
Supplement: Supplementary file 1 [file mmc1.docx]

# Supplementary Material

| **Table S1**  Adjusted, categories of care by the British Association of Perinatal Medicine (The British Association of Perinatal Medicine, 2011). | |
| --- | --- |
| **Intensive care** | **Day of surgery, day of death.**  **Any day receiving any of the following:**  Mechanical respiratory support via a tracheal tube  Presence of an umbilical arterial or venous line  Presence of a peripheral arterial line  Insulin infusion  Presence of a chest drain  Exchange transfusion  Therapeutic hypothermia  Prostaglandin infusion  Presence of a Replogle tube  Presence of an epidural catheter  Presence of a silo for gastroschisis  Presence of an external ventricular drain  Dialysis (any type) |
| **High-dependency care** | **Any day where an infant does not fulfil the criteria for intensive care**  **Any day receiving any of the following:**  Non-invasive respiratory support (e.g., nasal CPAP, SIPAP, BIPAP, HHFNC)  Parenteral nutrition  Continuous infusion of drugs (except prostaglandin and /or insulin)  Presence of a central venous or long line (PICC)  Presence of a tracheostomy  Presence of a urethral or suprapubic catheter  Presence of a trans-anastomotic tube following oesophageal atresia repair  Presence of NP airway/nasal stent  Observation of seizures / CF monitoring  Barrier nursing  Ventricular tap |
| **Special care** | **Any day where an infant does not fulfil the criteria for intensive or high-dependency care and requires any of the following:**  Oxygen by nasal cannula  Feeding by nasogastric, jejunal tube or gastrostomy  Continuous physiological monitoring  Care of a stoma  Presence of an IV cannula  Receiving phototherapy  Special observation of physiological variables at least 4 hours |

Note*:* Abbreviations: BIPAP, Bilevel Positive Airway Pressure; CF monitoring, Cystic Fibrosis Monitoring; CPAP, Continuous Positive Airway Pressure; HHFNC, Heated Humidified High-Flow Nasal Cannula; NP airway, Nasopharyngeal Airway; PICC, Peripherally Inserted Central Catheter; SIPAP, Synchronized Intermittent Positive Airway Pressure.

| **Table S2**  RN staffing guidelines from the British Association of Perinatal Medicine (The British Association of Perinatal Medicine, 2022). | |
| --- | --- |
| **Infant Acuity level** | **Infant hours present : RN hours required** |
| Intensive care | 1 : 1 |
| Hight dependency care | 1 : 0.5 |
| Special care | 1 : 0.25 |
| Note: Intensive care = Complex, high risk infants; Hight dependency care = moderate risk infants; Special care =stable, low risk infants.  Abbreviation: RN, registered nurse. | |

| Table S3  Hours of nursing staff per NICU, shift and staff category. | | | | | | |
| --- | --- | --- | --- | --- | --- | --- |
| NICU Level  Shift type | **RN/RN+** hours (%) | **NA** hours (%) | **Staffing, total** hours (%) | **RN+** hours (%) | **RN** hours (%) | **Coord.** hours |
| Level 4 |  |  |  |  |  |  |
| Day (7h) | 4620.4 (53.8) | 3961.3 (46.2) | 8581.6 (100.0) | 3242.3 (70.2) | 1378.1 (29.8) | 725.3 |
| Evening (7h) | 4294.0 (51.4) | 4059.7 (48.6) | 8353.7 (100.0) | 2860.7 (66.6) | 1433.3 (33.4) | 2.5 |
| Night (10h) | 6003.6 (48.9) | 6262.2 (51.1) | 12265.8 (100.0) | 5094.9 (84.9) | 908.7 (15.1) | 0.0 |
| Total | 14917.9 (51.1) | 14283.2 (48.9) | 29201.1 (100.0) | 11197.9 (75.1) | 3712.1 (24.9) | 727.8 |
| Level 3 |  |  |  |  |  |  |
| Day (7h) | 3051.3 (38.5) | 4872.9 (61.5) | 7924.2 (100.0) | 2219.3 (72.7) | 831.9 (27.3) | 476.4 |
| Evening (7h) | 3028.6 (39.6) | 4613.7 (60.4) | 7642.3 (100.0) | 2086.1 (68.9) | 942.5 (31.1) | 0.0 |
| Night (10h) | 4663.4 (39.7) | 7091.5 (60.7) | 11754.9 (100.0) | 3172.5 (68.0) | 1490.9 (32.0) | 0.0 |
| Total | 10743.3 (39.3) | 16578.2 (60.7) | 27321.4 (100.0) | 7477.9 (69.8) | 3265.4 (21.2) | 476.4 |
| Level 2 |  |  |  |  |  |  |
| Day (7h) | 3174.1 (48.7) | 3346.6 (51.3) | 6520.7 (100.0) | 1173.4 (37.0) | 2000.7 (63.0) | 663.7 |
| Evening (7h) | 3234.5 (49.3) | 3320.3 (50.7) | 6554.8 (100.0) | 1587.4 (49.1) | 1647.1 (50.9) | 56.1 |
| Night (10h) | 4951.4 (48.5) | 5257.9 (51.4) | 10201.9 (100.0) | 3851.5 (78.0) | 1099.8 (22.2) | 0.0 |
| Total | 11360.0 (48.8) | 11917.4 (51.2) | 23277.4 (100.0) | 6612.4 (58.3) | 4747.6 (41.7) | 719.8 |
| Total h  all units | 37021.2 (46.4) | 42778.7 (53.6) | 79799.9 (100.0) | 25288.2 (68.3) | 11733.1 (28.9) | 1923.9 |
| Note: Level 4 units provided full intensive care (including neonatal surgery) for the most medically complex and critically ill infants.  Level 3 units provide care for medically complex and critically ill infants from gestational weeks 26.  Level 2 units care for stable or moderately ill infants at ≥32 weeks of gestation.  Abbreviations: Coord, coordinator; NICU, neonatal intensive care unit; RN, registered nurse; RN+, registered nurse with a specialist education; NA, nursing assistant. | | | | | | |


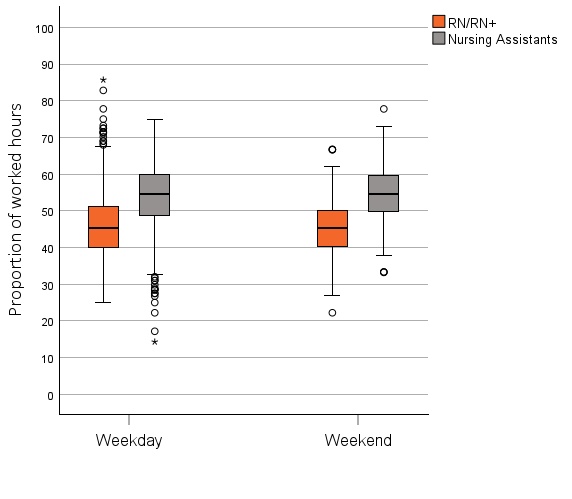


**Fig. S1**

Distribution of staff hours by staff category and type of day.

Whiskers extend to 1.5 × IQR from the box. Circles (o) denote mild outliers, and stars (*) represent extreme outliers.

Abbreviations: RN, registered nurse; RN+, registered nurse with a specialist education.

**
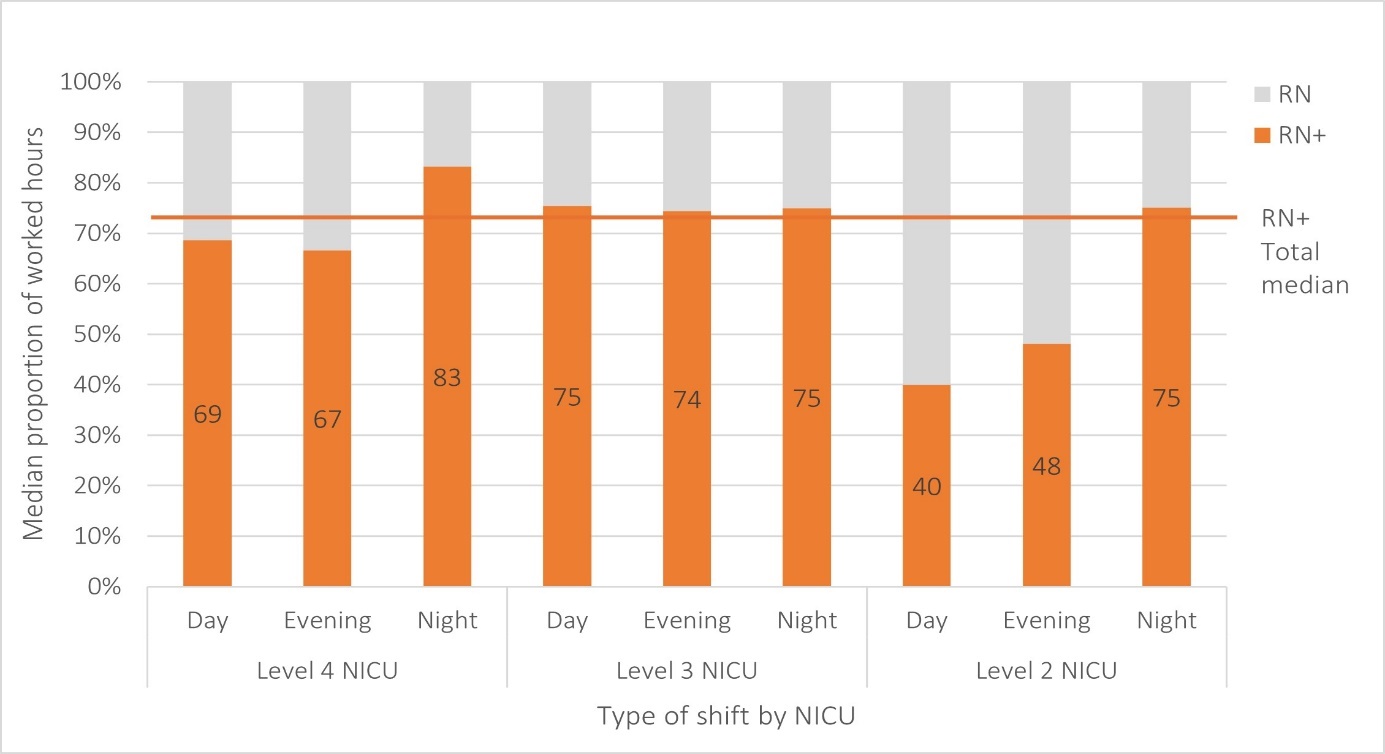
Fig. S2**

Median proportion of registered nurse hours with specialist education, by NICU and shift type.

*Note:* Level 4 units provided full intensive care (including neonatal surgery) for the most medically complex and critically ill infants.

Level 3 units provided care for medically complex and critically ill infants from gestational week 26.

Level 2 units cared for stable or moderately ill infants at ≥32 weeks of gestation.

Abbreviations: RN, registered nurse; RN+, registered nurse with a specialisation

| **Table S4**  In-hospital days by infant acuity category and NICU. | | | | |
| --- | --- | --- | --- | --- |
| **Infant acuity category**  In-hospital days, n (%) | **Level 4 NICU** | **Level 3 NICU** | **Level 2 NICU** | **Total** |
| Intensive care | 520 (42.0) | 289 (17.1) | 58 (3.3) | 867 (18.6) |
| High dependency care | 590 (47.7) | 693 (41.1) | 660 (37.7) | 1943 (41.6) |
| Special care | 128 (10.3) | 705 (41.8) | 1031 (58.9) | 1864 (39.9) |
| Total | 1238 (100.0) | 1687 (100.0) | 1749 (100.0) | 4674 (100.0) |
| Note: Level 4 units provided full intensive care (including neonatal surgery) for the most medically complex and critically ill infants.  Level 3 units provided care for medically complex and critically ill infants from gestational week 26.  Level 2 units cared for stable or moderately ill infants at ≥32 weeks of gestation.  Abbreviation: NICU, neonatal intensive care unit. | | | | |

| **Table S5**  Registered nurse provision ratio, all units, per shift-type for weekdays and weekends. | | | |
| --- | --- | --- | --- |
| Type of shift | **Weekday**  (240 shifts per shift type)  median RNPR (IQR) | **Weekend**  (92 shifts per shift−type)  median RNPR (IQR) | P−value weekend vs. weekday ^a^ |
| Day | 0.79 (0.35) | 0.74 (0.31) | 0.015 |
| Evening | 0.77 (0.28) | 0.75 (0.28) | 0.218 |
| Night | 0.76 (0.27) | 0.79 (0.30) | 0.796 |
| Note: Values below 1.0 indicate a lower−than−recommended presence of RNs, as per the BAPM guidelines, relative to infants staffing needs. ^a^ Independent sample test; Mann−Whitney U.  Abbreviations: RN, registered nurse; RNPR, registered nurse provision ratio. | | | |

# References

The British Association of Perinatal Medicine. (2011). *Categories of Care*. https://www.bapm.org/resources/34-categories-of-care-2011

The British Association of Perinatal Medicine. (2022). *Service and Quality Standards for Provision of Neonatal Care in the UK*. https://hubble-live-assets.s3.amazonaws.com/bapm/file_asset/file/1494/BAPM_Service_Quality_Standards_FINAL.pdf
